# Combination of Shengji ointment and bromelain in the treatment of exposed tendons in diabetic foot ulcers: study protocol for a non-blind, randomized, positive control clinical trial

**DOI:** 10.1186/s12906-023-04128-z

**Published:** 2023-10-10

**Authors:** Yang Zhao, Xinyue Dai, Xu Sun, Zhaohui Zhang, Hongyang Gao, Rui Gao

**Affiliations:** 1 Xiyuan Hospital of China Academy of Chinese Medical Sciences, Haidian District, Beijing, China; 2 NMPA Key Laboratory for Clinical Research and Evaluation of Traditional Chinese Medicine, Beijing, China; 3 National Clinical Research Center for Chinese Medicine Cardiology, Beijing, China; 4 China Academy of Chinese Medical Sciences, Beijing, China; 5 The Second Affiliated Hospital of Tianjin University of Traditional Chinese Medicine, Tianjin, China

**Keywords:** Administration, topical, Bromelain, Diabetic foot, Herbal medicine, Medicine, Chinese Traditional, Ointment, Tendon injuries

## Abstract

**Background:**

Diabetic foot ulcers often affect tendon tissue. Consequently, the infection may spread proximally along the tendon, leading to amputation or even the death of patients. Exposed, degenerated, and necrotic tendons are key factors affecting the healing of diabetic foot ulcers. The effective treatment of the tendon involvement may positively affect the prognosis. In clinical practice, treatment with Shengji ointment and bromelain induces islands of granulation tissue on the denatured tendon surface, which gradually grows and merges. Ideally, the exposed tendon is covered entirely by granulation tissue.

This trial aims to assess the effect of a combined treatment regime of Shengji ointment, which has been shown to regenerate muscle tissue and pineapple protease in preventing the loss of function and amputation caused by tendon necrosis. This trial will provide high-quality evidence for the effectiveness of this combination in healing diabetic ulcers with tendon necrosis.

**Methods:**

The sample size will be 180 patients who will be randomly assigned 1:1 to a treatment group (90 patients) using Shengji ointment combined with bromelain and a control group (90 patients) using hydrocolloid dressing. Both groups will continue their conventional treatments, such as blood glucose and blood pressure medication, lipid regulation, antiplatelets, and others. The primary outcome will be the wound coverage with granulation tissue. Secondary outcomes will be the wound healing rate, amputation extent (where needed), time to granulation, and the Maryland Foot Score. Other efficacy outcomes will be the time to debridement of necrotic tendon tissue and granulation tissue score.

**Discussion:**

This study will treat patients with diabetic foot ulcers with exposed, degenerated, and necrotic tendons with Shengji ointment and bromelain. The trial aims to promote regeneration and healing, to preserve the limb and its function, and to develop a comprehensive and effective protocol that can be applied to promote the healing of exposed tendons in diabetic foot wounds.

**Trial registration:**

ChiCTR2000039327; date of registration: 2020-10-23.

**Supplementary Information:**

The online version contains supplementary material available at 10.1186/s12906-023-04128-z.

## Administrative information

Note: the numbers in curly brackets in this protocol refer to SPIRIT checklist item numbers. The order of the items has been modified to group similar items (see http://www.equator-network.org/reporting-guidelines/spirit-2013-statement-defining-standard-protocol-items-for-clinical-trials/).

**Table:** 

Title {1}	Combination of Shengji ointment and bromelain for the treatment of exposed tendons in diabetic foot ulcers: Study protocol for a non-blind, randomized, positive control clinical trial
Trial registration {2a and 2b}.	ChiCTR2000039327
Protocol version {3}	V3.0
Funding {4}	National key research and development plan funding (2019YFC1709300)
Author details {5a}	Yang Zhao^145^, Xinyue Dai^2^, Xu Sun^3^, Zhaohui Zhang^3^, Hongyang Gao ^145^, Rui Gao^145^ ^1^Xiyuan Hospital China Academy of Chinese Medical Sciences ^2^China Academy of Chinese Medical Sciences ^3^ s Affiliated Hospital of Tianjin University of TCM ^4^NMPA Key Laboratory for Clinical Research and Evaluation of Traditional Chinese Medicine ^5^National Clinical Research Center for Chinese Medicine Cardiology
Name and contact information for the trial sponsor {5b}	Rui Gao, Xiyuan Hospital China Academy of Chinese Medical Sciences, Haidian District, Beijing, China.
Role of sponsor {5c}	Study design, writing of the report, and the decision for publication.

## Background and rationale {6a}

A diabetic foot is defined as the destruction of skin and deep tissues distal from the ankle joint in patients with diabetes, often combined with infection and/or peripheral arterial occlusive disease of varying degree in the lower limbs and, in severe cases, involving muscle and bone tissue [1]. Diabetic foot is one of the most common and serious chronic complications of diabetes and a frequent cause of disability and death [2]. It is estimated that a limb is amputated every 20 s in patients with diabetes worldwide [3]. Diabetic foot ulcers often extend deep into tissue and the underlying tendons, and the infection can spread proximally along the tendon, eventually leading to amputation or death [4]. Exposed, degenerated, and necrotic tendons impair the healing of diabetic foot ulcers [5], and the effective treatment of these tendon injuries improves the prognosis of wound healing [6].

The *International Diabetic Foot Working Group* guidelines emphasize that the management of diabetic foot ulcers requires a multidisciplinary and comprehensive approach [7]. In clinical Traditional Chinese Medicine practice, denatured but still vital tendon surfaces are covered with patches of new granulation tissue that gradually grow and eventually merge with each other when treating diabetic ulcers with a combination of Shengji ointment and bromelain [8].

In Traditional Chinese Medicine (TCM), treatment according to the method of Hua Fu Sheng Ji entails bromelain, which targets liquefied necrotic tendon and fascia, removes necrotic tissue, and prevents further putrefaction, thereby providing a clean wound bed that promotes healing in diabetic ulcers [9]. The combination of bromelain with Shengji ointment uses the synergistic effects of the substances and promotes the in situ regeneration of granulation tissue and epithelial tissue. It has been shown that the content of total protein, total amino acid, and lysozyme per unit of wound exudate increases significantly over different periods of time under this treatment, providing the nutrients for wound healing [10]. This may result in a reversal of the denaturation of the tendon tissue and stimulates the growth of granulation tissue that starts to cover tendon tissue, which may allow to retain parts of the tendon and to preserve foot function to a certain extent.

Previous animal studies [11] have shown that the application of bromelain combined with Shengji ointment can target and remove necrotic tendon tissue, promote tissue regeneration, and realize tissue repair in situ. The healing rate was 60%, and the efficacy rate was 94.3%. By changing the pH-value and lysozyme content of the wound, the method may liquefy the necrotic tendon and fascia, control infection, and stop decay. The TCM ointment Shengji mainly focuses on the growth of muscle and skin by providing amino acids, proteins, and other substances needed for wound repair and stimulating epidermal growth. The combination of Shengji with bromelain can accelerate tissue growth and promote wound healing. Our preliminary research [National Natural Science Foundation Project (30873270): Mechanism Research on the Effect of Decomposing Rot and Promoting Muscle Growth on Diabetic Foot Tendon Necrosis [12] has found that some degenerated tendon tissue recovered after treatment, and granulation tissue grew to cover the tendon tissue, retaining parts of the tendons and preserving foot function to a certain extent, which we called the “Jin Zhi Xue Hua” phenomenon. Later, based on further research [National Natural Science Fund Project (81573972) [13], we found that the creation of a steady-state microenvironment promoted the perfusion of tendons during the healing of diabetic foot ulcers] and concluded that “the method of Hua Fu Sheng Ji can promote a stable microenvironment in diabetic foot ulcers.” It is of great significance to explore the underlying mechanisms to improve diabetic foot ulcer repair and to enrich the theory of topical treatment in TCM.

This clinical trial will follow a multicenter, randomized, positive control design aimed at assessing the combination treatment’s efficacy in promoting tendon healing and preserving both the limb and its function in patients with Wagner grade 3–4 diabetic foot ulcers and tendon exposure.

The trial aimed to (a) investigate the effect of Shengji ointment and pineapple protease, used for muscle regeneration and healing, as therapeutic drugs in preventing amputation necessitated by tendon degeneration and necrosis, thereby preserve limb function, and to (b) develop high-quality evidence and a comprehensively effective plan for promoting the healing of exposed tendons in diabetic foot ulcers.

### Objectives {7}

The objectives of this trial are as follows:

1. Create high-quality evidence on the effects of the treatment of diabetic ulcers with a combination of Shengji ointment;
2. Evaluate the efficacy and safety of the combination treatment;
3. Develop a generalizable TCM treatment plan for diabetic foot ulcers with tendon exposure.

### Trial design {8}

This study was a multicenter, non-blind, randomized, positive control trial.

## Methods: participants, interventions, and outcomes

### Study setting {9}

The diagnostic criteria for diabetic foot refer to.

1. The Technical Guiding Principles for Clinical Research of New Traditional Chinese Medicine for Diabetic Foot (Draft for Soliciting Opinions [14]) and the Guidelines for Diagnosis and Treatment of Diabetic Foot in China (2019 Edition) [15] issued by the Drug Evaluation Center of the State Drug Administration;
2. Diabetic foot grading standard: Wagner grading.

### Eligibility criteria {10}

#### Inclusion criteria

Patients who meet the following criteria will be included:

1. Diagnostic criteria for diabetic foot disease and Wagner grade 3–4 ulcers with exposed tendon tissue;
2. Age between 18 and 85 years;
3. Fasting blood glucose ≤ 10 mmol/L;
4. Targeted ulcer debridement area between 1 and 20 cm^2^ (for patients with multiple lesions, the largest ulcer will be the target lesion);
5. An ankle-brachial index ≥ 0.5 on the side of the limb with the ulcer;
6. The ulcer has blood, pus, or sticky secretion;
7. Voluntary participation and signing of an informed consent form.

#### Exclusion criteria

Patients who meet any of the following criteria will be excluded:

1. Skin ulcer caused by electrical, chemical, or radiation injury, tumors, varicose veins, or other reasons; or malignant lesions within the ulcer;
2. Severe clinical infection indicated by cellulitis, fever, elevated white blood cell count, bacterial culture, or increased (high-sensitivity) C-reactive protein levels;
3. Severe uncontrollable hypertension with systolic blood pressure ≥ 160 mmHg or diastolic blood pressure ≥ 110 mmHg;
4. Serum albumin levels < 28 g/L;
5. Hemoglobin < 90 g/L;
6. Platelet count < 50 × 10^9^/L;
7. Severe heart, liver, or kidney injury, in case of medical treatment that may seriously affect the safety and treatment;
8. Pregnancy, family planning, or breastfeeding women;
9. Cognitive dysfunction preventing fully informed consent;
10. Allergic disposition or allergic to the ingredients of the treatment under investigation and reference drugs;
11. Participation in other clinical trials during the past one month;
12. In the judgment of the researcher, inability to complete the trial or comply with its requirements.

### Who will take informed consent {26a}

Before a patient is enrolled in this trial, the researcher is responsible for explaining the purpose, nature, procedure, possible benefits, and risks of their participation to them or their designated representative in written form. Patients shall be informed that they have the right to withdraw from the study at any time. A written informed consent form must be given to each patient before enrollment such that they can confirm that they understand and agree with their participation. Informed consent forms must be voluntarily signed before the patients are enrolled in the trial. The informed consent form will be kept as one of the original materials for the trial.

### Interventions

#### Explanation for the choice of comparators {6b}

As a commonly used dressing for diabetic foot wounds, the hydrocolloid dressings are safe, reliable, effective, and superior in the treatment of diabetic foot ulcers and other chronic difficult-to-treat wounds. The hydrocolloid dressing was determined to be the best comparator as a positive control.

#### Intervention description {11a}

Both the intervention and control groups will receive routine medical and surgical treatment (blood sugar control, blood pressure reduction, lipid regulation, antiplatelet medication, debridement, and others).

In the intervention group, bromelain powder will be applied to the exposed tendon and necrotic tissue, and the wound will be covered with Shengji ointment. In the control group, a hydrocolloid dressing will be used to cover the wound.

##### Treatment 1: Shengji ointment

- Size: 30 g/box.
- Formulation: Ointment.
- Usage and dosage: For external use. The ointment will be spread on skimmed cotton and applied to the affected area.
- Route of administration: Topical.
- Frequency of administration: Once every 24 h.
- Treatment course: Four weeks.
- Manufacturer: Tianjin Darentang Jingwanhong Pharmaceutical Co., Ltd, Tianjin, Tianjin, China.

##### Treatment 2: Pineapple protease (bromelain)

- Specification: 10,000 units.
- Formulation: Tablet.
- Dosage: For external use. It will be applied to exposed tendons and areas with necrotic tissue.
- Route of administration: Topical.
- Frequency of administration: Once every 24 h.
- Treatment course: Four weeks.
- Manufacturer: Shantou Olive Pharmaceutical Co., Ltd., Shantou City, Guangdong, China.

##### Active control: Comfeel® Plus wound dressing

- Size: 25 g/piece.
- Formulation: Hydrocolloid dressing.
- Usage and dosage: Apply Comfeel® dressing to the ulcer. Ensure the dressing height is at the level of the surrounding skin. Then, apply a layer of dressing over the ulcer and surrounding area.
- Manufacturer: Coloplast Group, Humblebaek, Denmark.
- Route of administration: Topical.
- Frequency of administration: Once every 24 h.
- Course of treatment: Four weeks.

#### Criteria for discontinuing or modifying allocated interventions {11b}

In enrolled patients who withdraw from the trial without completing the clinical protocol for any reason during the trial, two conditions will be distinguished: researcher-determined discontinuation and voluntary withdrawal.

##### I. Termination of a patient’s participation based on the researcher’s decision

The researchers may decide to withdraw enrolled subjects from the study when they establish that they are not suitable to continue the study for any of the following reasons:

1. During the trial, clinical endpoints (such as the need for surgery) are reached.
2. The patient undergoes surgical treatment other than debridement of the ulcer site.
3. During the clinical trial, patients experience complications or physiological changes that make it inappropriate for them to continue to receive treatment within the trial.
4. Use of other prohibited treatments or drugs that affect the assessment of the efficacy and safety of the intervention.
5. If serious adverse events occur during the trial, the researcher may decide whether to suspend the trial.
6. Poor compliance of the patients that results in the failure of 80% of the prescribed dosage of topical drugs (except cured patients) or the failure of 120% of the prescribed dosage.

Subjects who will be withdrawn from the trial will undergo a final visit to evaluate each efficacy indicator and to complete a safety assessment.

##### Patients who voluntarily withdraw from the trial

Every patient has the right to withdraw from the trial in accordance with the Good Clinical Practice (GCP) and informed consent. “Withdrawal” also refers to the loss of a patient after he/she no longer receives the treatment and assessment of the intervention, although he/she does not explicitly propose withdrawal from the study.

1. If the intervention or control is found to be ineffective, the patient may be unwilling to continue the trial. If the patient proposes to withdraw from the trial to the researcher before returning to the routine treatment.
2. Patients who, due to various other reasons, are unwilling or unable to continue the clinical trial and terminate the trial by offering to withdraw from the trial to the researcher.
3. Although the patients do not explicitly propose withdrawing from the study, they are no longer followed up and are thus lost.

Researchers will aim to learn as much as possible about the reasons for patients’ withdrawal and record them. For example, the perceived curative effect may not be good; patients may not tolerate some adverse reactions; patients may be unable to continue the clinical trial for various reasons, e.g., economic factors; or loss of follow-up without explanation.

##### II. Suspension/termination criteria for the whole trial:

Suspension/termination of the trial means that the entire clinical trial is not completed in accordance with the protocol. The purpose of a trial suspension/termination is to protect patients’ rights and interests, to ensure the quality of the trial, and to avoid unnecessary economic losses.

This trial may be discontinued for the following reasons:

1. When serious safety problems occur during the trial and the researcher believes that the safety of the patients may be compromised, or when the treatment is found to be too poor or ineffective during the trial to have a clinical value.
2. It is difficult to evaluate the efficacy and/or safety of the treatment because major errors are discovered in the trial protocol or significant deviations in its implementation occur during the trial.
3. The researcher proposes suspension (for example, funding or management reasons, or others).

The researcher will notify the patient, organization responsible for the trial, and Ethics Committee and explain the reasons for discontinuing the trial. The responsible research unit shall notify the investigator, Ethics Committee, and Ministry of Science and Technology before suspending the clinical trial and state the reasons.

#### Relevant concomitant care permitted or prohibited during the trial {11d}

No additional Chinese or Western drugs related to the treatment of the ulcer (vasodilators, such as lipid microspheres prostaglandin injection, beraprost sodium, cilostazol, sagresol hydrochloride, nefuram, butalbital, and hexaketone cocaine, among others; antiplatelet drugs, such as aspirin and clopidogrel; anti-coagulant drugs, such as unfractionated heparin or low-molecular-weight heparin, and oral anti-coagulants; or TCM or proprietary Chinese medicine, topical antibiotics, trimethoprim/sulfamethoxazole, and others, which have the effect of muscle growth and act as astringents in the treatment of sores) should be used during the trial. The same applies to biological treatments (such as stem cell therapy, topical autologous platelet-rich plasma, maggot therapy, cell growth factors, chymotrypsin, and TCM or Chinese herbal tonics with similar functions as the trial treatment).

Drugs that are required to treat comorbidities may be continued. These drugs must be recorded in detail on the case report form, including the name of the drug, dosage, frequency, and duration of use.

Glucose-lowering drugs (oral antidiabetics and insulin) will be selected according to the patient’s blood glucose level at the time of enrollment and treatment regimen. Except for uncontrolled hyperglycemia, the type of glucose-lowering drug or insulin regimens of patients will remain unchanged, although the dosage will be adjusted according to blood glucose levels. Blood glucose levels and glucose-lowering drug doses will be recorded in a timely and detailed manner on the case report form. Patients whose blood glucose levels cannot be normalized within a week will be discontinued from the trial.

### Outcomes {12}

#### Primary outcome

The wound coverage with granulation tissue will be the primary outcome. The development of granulation tissue will be assessed as follows:

$$\text{Wound coverage rate} = \text{The wound area covered by granulation tissue}\, (\text{mm}^{2})/\text{Whole wound area}\, (\text{mm}^{2}) \times 100\%$$

We will use three-dimensional scanning to obtain the wound surface topography and the inSight® platform (eKare, Inc., Fairfax, VA, USA) to identify the different tissue types and to measure the areas covered by them.

#### Secondary outcomes

As secondary outcomes, we will determine the wound healing rate, amputation rate and extent, granulation time, and Maryland Foot Score.

1. Wound healing rate = (Original wound area - unhealed wound area)/Original wound area ×100%.
2. Amputation extent: A extensive amputation refers to an amputation above the ankle, while a limited amputation refers to an amputation below the ankle. The amputation level will be identified in a multidisciplinary team of vascular surgeons and orthopedic foot and diabetes specialists according to the results of the lower limb angiography.
3. Granulation time (days): The period of time within which new granulation tissue appears within the wound.
4. The Maryland Foot Score will be determined to evaluate foot function, including the presence or absence of pain. The maximum score is 100 points, > 89 points indicate excellent function, 75–89 points good function, 50–74 average, and < 50 points poor function.

#### Other indicators of treatment efficacy

We will also record the following parameters:

Clearance time (days): The period from enrollment (day 0) to the complete clearance of degenerated and necrotic tendon tissue.

Granulation tissue score: Evaluation of scores at enrollment (day 0), visit 2 (week 2), and at the end of the trial (week 4).

#### Participant timeline {13}

The time schedule of the enrollment, interventions, assessments, and visits for the participants in this trial is shown in Table 1.

**Table 1:** Study time-period

	STUDY PERIOD			
	Enrolment	Allocation	Post-allocation	Close-out
TIMEPOINT	-1 day	0 day	After 14 days	After 28 days
ENROLLMENT:				
Eligibility screen	●			
Informed consent	●			
Allocation		●		
INTERVENTIONS:				
Treatment group: Shengji ointment and Pineapple protease (bromelain)		●	●	●
Control group: Comfeel® Plus wound dressing		●	●	●
ASSESSMENTS:				
Demographic data	●			
Medical history and allergy history	●			
Vital signs		●	●	●
Physical examination		●	●	●
Blood routine + C- reactive protein		●		●
Urine routine		●		●
Routine stool + occult blood		●		●
Liver function		●		●
Renal function		●		●
Electrocardiogram		●		●
Urine pregnancy test		●		
Wound coverage rate		●	●	●
Wound healing rate		●	●	●
Amputation extent			●	●
Granulation time			●	●
Maryland Foot Score		●	●	●
Clearance time			●	●
Granulation tissue score		●		●
Adverse events		●	●	●

#### Sample size {14}

The sample size was calculated using Pass 11.0 software (NCSS Statistical software https://www.ncss.com/software/pass). Based on a preliminary review of the literature and the clinical practice and experience of the Second Affiliated Hospital of Tianjin University of Traditional Chinese Medicine in the treatment of diabetic foot ulcers, the control group is expected to have an effectiveness of 50%, and the treatment group of 71%. Postulating a patient ratio of 1:1 between groups and an alpha of 0.05 on both sides, 72 patients would be required in each group. Assuming a 20% loss to follow-up, we calculated a number of 90 patients in each group and a total of 180 patients as the target sample size.

#### Recruitment {15}

The patients will be followed up at four hospitals; the Second Affiliated Hospital of Tianjin University of Traditional Chinese Medicine (80 patients), Affiliated Hospital of Liaoning University of Traditional Chinese Medicine (40 patients), Affiliated Hospital of Shanxi University of Traditional Chinese Medicine (40 patients), and Tianjin Binhai New Area Hospital of Traditional Chinese Medicine (20 patients).

Assignment of interventions: random allocation.

#### Sequence generation {16a}

Using a central random system and minimization algorithm, the allocation probability of the target group will be calculated for each enrolled patient for them to be assigned to the most suitable treatment group and ensuring a balance of control factors between the groups. The controlling factors are (1) ulcer location (plantar, dorsum of foot, or toe), and (2) ABI evaluation index (> 0.7, and ≤ 0.7).

#### Concealment mechanism {16b}

None.

### Data collection and management

#### Data management {19}

1. Research medical records.

Since most outpatient medical records at China’s hospitals are kept by the patients themselves, the case report form will be designed specifically for this trial to preserve first-hand information. The research medical records are the original documents of the trial patients, which are properly kept by each trial center.

2 Electronic data management.

The electronic case report form is created using the clinical trial data management system (eCDMS3.0) at Xiyuan Hospital developed by the Chinese Academy of Traditional Chinese Medicine, and the data will be collected and managed online.

1. Full analysis set: This refers to patients receiving the allocated treatment at least once and having data on at least one post-treatment primary outcome measurement. For patient data that fail to observe the whole treatment process, the data from the last observation will be carried forward to the final last observation carried forward.
2. Per protocol set: Good compliance (80% ≤ treatment compliance ≤ 120%), not taking prohibited medications during the study, and no serious violation of the trial protocol.
3. Safety set: All patients who are randomized and use the trial treatment at least once.

### Statistical methods for primary and secondary outcomes {20a}

1. Statistical description.
  1. Detailed description of cases of withdrawal and censoring, including the time and reason for censoring.
  2. Descriptive statistics: Based on their distribution, data will be presented as either the mean and standard deviation, or the median with maximum, minimum, and confidence interval, or the number and frequency (%), among others.
2. Statistical inference method.
  1. Measurement data: The paired t-test, analysis of variance, the rank-sum test, signed rank-sum test, and the median test will be used.
  2. Counting data: The chi-square test, Fisher exact test, and others will be used; grade data will be analyzed using the ridit test and the Cochran–Mantel–Haenszel test.
  3. Primary outcome analysis: Per protocol set analysis and full analysis set analysis will be performed. The Cochran-Mantel-Haenszel test will be used for multicenter counts and analysis of covariance for measures. For the confounding factors that are difficult to control or are uncontrolled before allocation, the least-squares means of the analysis of covariance and their 95% confidence limit or logistic regression will be used as covariates to determine the between-group efficacy.
  4. Two-sided difference tests will be used to assess statistical differences, and a difference with a p-value of ≤ 0.05 will be considered to be statistically significant.

### Oversight and monitoring

#### Quality control

Researcher authorization: All researchers are allowed to enter the trial and to conduct trial operations within the scope of authorization only after receiving training and authorization from the Principal Investigator.

Clinical monitoring: This clinical trial will be supervised by a clinical research auditor, who instructs the researchers to conduct the clinical trial in accordance with the trial protocol and GCP.

#### Quality assurance

Researcher training: Before the start of the trial, the principal investigation unit is required to train all researchers involved in the trial and others associated with the trial on the GCP and the trial protocol.

#### Standard operating procedures

All researchers must strictly follow the standard operating procedures provided for this trial.

#### Auditing

The sponsor is responsible for auditing the trial and providing proof of audit to the Principal Investigator.

#### Adverse event reporting and harms {22}

The adverse events, adverse reactions, and serious adverse events in this study will be recorded. The safety assessment will include the recording and analysis of.

1. All adverse events (including symptoms, signs, etc.);
2. Liver and kidney function, hematuria; stool routine, and electrocardiogram;
3. Pregnancy tests in women as indicated.

## Discussion

The exposure, degeneration, and necrosis of tendons are key factors affecting the healing of diabetic foot ulcers, and the successful treatment of the tendon injury affects prognosis. Clinical practice has shown that Shengji ointment combined with bromelain treatment can induce granulation islands to form on the denervated but not yet inactive tendons. These islands gradually grow together, eventually covering the previously exposed tendon with granulation tissue to heal the wound.

This multicenter, randomized, positive control trial will evaluate the clinical effects, validate the efficacy and safety of this combined treatment with the aim of developing a generalizable TCM treatment plan for diabetic foot ulcers with exposed tendons, and provide high-quality evidence for its clinical applications.

## Trial status

Protocol version number and date: Version 3.0, 23 Aug 2020.

The study was registered at the Chinese Clinical Trials Registry on 23 Oct 2020, registration number: ChiCTR2000039327. Recruitment was started on 1 Dec 2020 and is expected to end in December 2022.

### Supplementary Information

**Additional file 1. **Standard operating procedure for wound photographing.

**Additional file 2. **Standard operating procedure for wound debridement and dressing change.

**Additional file 3. **Maryland Foot Score evaluation standard.

**Additional file 4. **Scoring of granulation tissue growth in wounds.

**Additional file 5. **Ethical approval document.

**Additional file 6. **Copy of the original funding documentation.

**Additional file 7. **SPIRIT 2013 Checklist: Recommended items to address in a clinical trial protocol and related documents*.

## Data Availability

Not applicable.

## Abbreviations

GCP: Good Clinical Practice
TCM: Traditional Chinese Medicine
