# Supplementary material for: Combination of Shengji ointment and bromelain in the treatment of exposed tendons in diabetic foot ulcers: study protocol for a non-blind, randomized, positive control clinical trial
Source: BMC Complement Med Ther. 2023 Oct 10;23:359. doi: 10.1186/s12906-023-04128-z (PMC10565983; doi:10.1186/s12906-023-04128-z)
Supplement: Supplementary file 1 — Additional file 1. Standard operating procedure for wound photographing. [file 12906_2023_4128_MOESM1_ESM.pdf]

Additional file 1: Standard operating procedure for wound photographing

Wound photography is an important means to objectively and accurately show the surface of patients' wounds. It is also one of the objective parameters in quality control and a method of data preservation in clinical research. Therefore, we developed this standard operating procedure for the taking of photos of diabetic foot ulcers to ensure adequate documentation that meets minimal standards, can help in the evaluation of the results, and improves the research quality on this subject.

Specific operating procedures are as follows:

1. Choose a neutral background or use a disposable dressing waste bag (blue) to cover a busy or messy background. This can also help to absorb the light and reflections to avoid changes in the wound color.

2. Use the same wound ruler throughout the entire observation. The patient's initials, number, and diagnosis (disease and classification) and the time, horizontal and vertical coordinates, scale, and color card of the photograph should be displayed on the wound ruler. The color card is set according to the black, yellow, and red powder of the wound, and the wound ruler should not cover the skin around the wound.

3. The patient should be in a comfortable position while you take pictures. All dressings should be removed before the photograph such that any bleeding, secretion, and the surrounding tissue of the ulcer with the exposed tendon can

be documented. After removing the dressing, routinely disinfect the wound surface, remove any surface secretion with dry cotton wool, and take photos before the debridement;

4. Operators should use cameras or mobile phones and avoid using any devices or software with decorative functions. The longitudinal axis of the mobile phone should be parallel to the long axis of the patient’s limb to ensure clarity and to maximize the photograph range. The photo size ratio is set to 12:9 (4:3).

5. The photos of each patient should be taken with the same equipment throughout the trial. The wound is placed in the center of the line-of-sight reference frame. The shooting angle and distance are consistent (the photograph is taken at an angle of 90 °to the vertical), to avoid any distortion. All photos should be taken according to the predefined observation times of the trial, and all files should keep the original images.

6. The photos should be sorted and stored in due time, and the photos in the camera and mobile phone should be backed up as soon as possible to avoid accidental deletion. Independent photographs should be established for each patient, and the documents should be managed according to the date. The treatment methods and any medication used at the time of the photographs, and the changes in the wound from before to after treatment should be recorded in detail.
