# Supplementary material for: Combination of Shengji ointment and bromelain in the treatment of exposed tendons in diabetic foot ulcers: study protocol for a non-blind, randomized, positive control clinical trial
Source: BMC Complement Med Ther. 2023 Oct 10;23:359. doi: 10.1186/s12906-023-04128-z (PMC10565983; doi:10.1186/s12906-023-04128-z)
Supplement: Supplementary file 3 — Additional file 3. Maryland Foot Score evaluation standard. [file 12906_2023_4128_MOESM3_ESM.pdf]

Additional file 3: Maryland Foot Score evaluation standard

**Evaluation content**	**Score**
**i Pain**	
Painless, including during exercise	45
Slight pain but no change in daily life or work	40
Slight pain, only minor changes in daily life or work	35
Moderate pain, activities of daily living decreased significantly	30
There is obvious pain during lighter daily life activities such as bathing and simple housework, and it is necessary to frequently take strong painkillers	10
Disabled, unable to work or go to shops	5
**ii Function**	
Walking distance	
Unlimited	10
The walking distance is slightly limited	8
The walking distance is moderately limited (2 or 3 blocks)	5
The walking distance is severely limited (1 block)	2
You can only move indoors	0
Stability	
Normal	4

Feeling weak-not really weak	3
Occasionally soften (once every 1-2 months)	2
Often soft	1
Need to use braces	0
<u>Auxiliary</u>	
No need	4
Walking stick	3
A cane	1
Wheelchair	0
<u>Limp</u>	
None	4
Mild	3
Moderate	2
Severe	1
Unable to walk	0
<u>Wear shoes</u>	
Freely	10
There are small obstacles	9
Shoes with flat belts	7
Orthopedic shoes Herzog	5
Padded shoes	2

No shoes	0
Go upstairs	
Normal	4
Need to help stair handrail	3
Use any other method	2
Not able	0
Requirements for the ground	
You can walk on any ground	4
There is a problem walking on the ground and hills in Stone	2
There is a problem walking on flat ground	0
Appearance	
Normal	10
Mild deformity	8
Moderate deformity	6
Severe deformity	0
Multiple deformities	0
Activity (ankle joint, subtalar joint, tarsal joint, toe joint) and contralateral comparison	
Normal	5
Mild decrease	4
Significantly reduced	2

Rigid	0
Note: Excellent, 90-100 points; Good, 75-89 points; Moderate, 50-74 points;<br><br>Poor, 0 – 49 points	
