# Supplementary material for: Combination of Shengji ointment and bromelain in the treatment of exposed tendons in diabetic foot ulcers: study protocol for a non-blind, randomized, positive control clinical trial
Source: BMC Complement Med Ther. 2023 Oct 10;23:359. doi: 10.1186/s12906-023-04128-z (PMC10565983; doi:10.1186/s12906-023-04128-z)
Supplement: Supplementary file 4 — Additional file 4. Scoring of granulation tissue growth in wounds. [file 12906_2023_4128_MOESM4_ESM.pdf]

Additional file 4: Scoring of granulation tissue growth in wounds

**Growth of wound granulation**	**score**
The granulation grows well, completely covers the wound, and its color is bright red	5 points
The granulation grows well, mostly covers the wound surface, and its color is bright red	4 points
The granulation grows well, covers more than 50% of the wound, and its color is bright red	3 points
Granulation growth is observed, covering 25%-50% of the wound	2 points
Granulation growth is observed and covers less than 25% of the wound surface.	1 point
No granulation growth is observed	0 points
