# Supplementary material for: Combination of Shengji ointment and bromelain in the treatment of exposed tendons in diabetic foot ulcers: study protocol for a non-blind, randomized, positive control clinical trial
Source: BMC Complement Med Ther. 2023 Oct 10;23:359. doi: 10.1186/s12906-023-04128-z (PMC10565983; doi:10.1186/s12906-023-04128-z)
Supplement: Supplementary file 5 — Additional file 5. Ethical approval document. [file 12906_2023_4128_MOESM5_ESM.pdf]

Additional file 5: Ethical approval document

Medical Ethics Committee of the Second Affiliated Hospital of Tianjin University of Traditional Chinese Medicine

Approval for ethical review

Ethical review approval number	2020-006-01
Project name	Combination of Shengji ointment and bromelain in the treatment of exposed tendons in diabetic foot ulcers: Study protocol for a non-blind, randomized, positive control clinical trial
Project source (clinical approval document)	Ministry of National Science and Technology (2019YFC1709303)
Sponsor	The Second Affiliated Hospital of Tianjin University of Traditional Chinese Medicine
Unit in charge of clinical research	The Second Affiliated Hospital of Tianjin University of Traditional Chinese Medicine
Clinical study participants	Affiliated Hospital of Liaoning University of Traditional Chinese Medicine, Affiliated Hospital of Shanxi University of Traditional Chinese Medicine, Tianjin

	Binhai New Area Hospital of Traditional Chinese Medicine		
The center undertakes professional	Surgery major of traditional chinese medicine	Principal investigator (applicant)	Zhang Chaohui
Categories of review	Review after initial examination	Investigation method	Conference review
Date of review	September 24, 2020		
Review location	Ethics Review Office of the Second Affiliated Hospital of Tianjin University of Chinese Medicine		
Review documents:			
Study protocol version number	V3.0	Solution version date	August 23, 2020
Version number of informed consent form	V2.0	Informed consent form version date	August 10, 2020
Others	Responses regarding ethical review of study subjects, investigator's brochure (v2.02020－0820), subject enrollment advertisement (v2.02020－0820), and description of modifications		
Review comments:			

<table>
<tr><td colspan="5">Following a review by the Ethics Committee, this study was conducted according to the approved clinical research program following the Measures for Ethical Review of Biomedical Research Involving People (2016) issued by the Ministry of Health, Good Laboratory Practice for Drugs (2020), Good Laboratory Practice for Medical Devices (2016) by the State Food and Drug Administration, WMA Declaration of Helsinki (2013) and CIOMS International Ethical Guidelines for Research Involving Human Health (2016), the guiding principles for ethical review of clinical trials of drugs (2010), and the ethical principles of the management norms for ethical review of clinical research of traditional Chinese medicine (2010).</td></tr>
<tr><td>Annual periodic follow-up review frequency</td><td>December</td><td colspan="3">Validity period: 23 September, 2021</td></tr>
<tr><td rowspan="2">Signature of the chairman
date</td><td>Ying Sinlin</td><td colspan="3" rowspan="2">The Second Affiliated Hospital of Tianjin University of Traditional Chinese Medicine
Medical Ethics Committee (Seal)</td></tr>
<tr><td>September 24, 2020</td></tr>
<tr><td>Contacts</td><td>Gu xufang</td><td>Contact by words.</td><td colspan="2">022-60637912</td></tr>
<tr><td colspan="5">Remarks information:
Please conduct the clinical study in accordance with the GCP principles and in accordance with the protocol approved by the Ethics Committee to protect</td></tr>
</table>

the health and rights of the subjects. Applicants are requested to complete clinical trial enrollment before study initiation.

Any changes to the clinical study protocol, informed consent forms, enrollment materials, and others, resulting from changes to the primary investigator during the study are requested to be submitted by the applicant for amendment review.

Serious adverse events: Please ensure that the applicants submit the serious adverse events report in a timely manner.

Request that the frequency of the annual/periodic follow-up review be as that set by the Ethics Committee and that the progress report be submitted by the applicant within 1 month of the deadline; The sponsor shall submit a summary report on the progress of the research of each center to the ethics committee of the group leader; Applicants are requested to submit a written report to the Ethics Committee in a timely manner when any condition occurs that may significantly affect the conduct of the trial or increase the risk to the subject.

When the study included subjects who did not meet the inclusion or exclusion criteria, meet the discontinuation of trials condition without the withdrawal of subjects from the study, gave incorrect treatment or dose administration, gave concomitant medications that is prohibited by the protocol, have a significant impact on the rights and interests/health of the subject, and

violate the GCP principles, the report of the violation is to be submitted by the sponsor/inspector/researcher.

Please ensure that the applicants who have suspended or prematurely terminated the clinical study have submitted the suspension/termination report in time.

Complete the clinical study and request the applicants to submit a final report.
