# Supplementary material for: Combination of Shengji ointment and bromelain in the treatment of exposed tendons in diabetic foot ulcers: study protocol for a non-blind, randomized, positive control clinical trial
Source: BMC Complement Med Ther. 2023 Oct 10;23:359. doi: 10.1186/s12906-023-04128-z (PMC10565983; doi:10.1186/s12906-023-04128-z)
Supplement: Supplementary file 6 — Additional file 6. Copy of the original funding documentation. [file 12906_2023_4128_MOESM6_ESM.pdf]

Additional file 6: Copy of the original funding documentation.

English translation file:

Ministry of National Science and Technology

Research project task book (No. 2019YFC1709303)

Project name	Combination of Shengji ointment and bromelain in the treatment of exposed tendons in diabetic foot ulcers: Study protocol for a non-blind, randomized, positive control clinical trial
Project affiliation	Evidence-based evaluation and curative effect mechanism of integrated traditional Chinese and western medicine treatment for diabetic foot the study
Project type	Research on Modernization of Traditional Chinese Medicine
Project lead institution	Xiyuan Hospital China Academy of Chinese Medical Sciences
Responsible institution	The Second Affiliated Hospital of Tianjin University

	of Traditional Chinese Medicine
Principal investigator	Zhang Chaohui
execution time	Dec 2019 – Dec 2021
